# Getting “Unstuck”: A Multi-Site Evaluation of the Efficacy of an Interdisciplinary Pain Intervention Program for Chronic Low Back Pain

**DOI:** 10.3390/healthcare4020033

**Published:** 2016-06-14

**Authors:** Timothy Clark, Jean Claude Wakim, Carl Noe

**Affiliations:** 1 Baylor Center for Pain Management, 3600 Gaston Ave, Wadley Tower, Suite 360, Dallas, TX 75246, USA; Jean.Wakim@BSWHealth.org; 2 Eugene McDermott Center for Pain Management—University of Texas Southwestern Medical Center, 1801 Inwood Avenue, Suite WA 7.5, Dallas, TX 75390, USA; Carl.Noe@utsouthwestern.edu

**Keywords:** low back pain, interdisciplinary treatment, effectiveness, biopsychosocial, outcome measures

## Abstract

Chronic low back pain is one of the major health problems in the U.S., resulting in a large number of years of disability. To address the biopsychosocial nature of pain, interdisciplinary pain programs provide integrated interventions by an interdisciplinary team in a unified setting with unified goals. This study examined outcomes of an interdisciplinary program located at two sites with different staff, yet with a unified model of treatment and documentation. Efficacy at the combined sites was examined by comparing standard measures obtained upon admission to the program with measures at completion of a 3–4 week long program for 393 patients with chronic low back pain (CLBP). Repeated measures included pain severity, pain interference, efficacy of self-management strategies, hours of activity, depression, ability to do ADLs, and physical endurance. All repeated measures differed at the *p* < 0.001 level, with large effect sizes (0.66–0.85). Eighty-two percent of graduates reported being “very much improved” or “much improved”. A second analyses provided evidence that treatment effects were robust across sites with no differences (<0.001) found on five of seven selected outcome measures. A third analysis found that number of days of treatment was correlated on three of seven measures at the <0.01 level. However, the amount of variance explained by days of treatment was under 5% on even the most highly correlated measure. These finding are consistent with previous research and explore short-term effectiveness of treatment across treatment sites and with variable duration of treatment.

## 1. Introduction

“Whenever I see my doctor for another visit; he seems to do the Michael Jackson moonwalk—I can feel him backing out of the room at the same time he is walking in”. Patient with chronic pain.

This patient’s (probably accurate) perception captures the frustration of both healthcare providers and patients facing chronic low back pain (CLBP). This subjective impression of being “stuck” is reflected in the continued suffering and disability despite the high cost of ongoing healthcare. As evidenced by recent reports by the Institute of Medicine [1,2], between $560 and $630 billion is spent annually on direct and indirect costs of chronic pain. These costs continue, or may in fact increase, despite advancements in pain medicine, in new pain medication, assessment of genotypes predicting medication response [3], and new surgical and interventional procedures and devices, among others. As the most common type of musculoskeletal pain, low back pain continues to be a primary source of disability, as evidenced in both emergency department and inpatient medical stays [4].

In an effort to better understand the problem of CLBP, some have suggested that the problem is more accurately understood and more effectively treated by use of a biopsychosocial model [5]. The biopsychosocial understanding of pain suggests that perceived pain and ensuing disability result from a complex array of factors interacting over time. This model suggests that success in the treatment of chronic pain is diminished by a simplistic focus on medical intervention for nociception. In fact, some recent literature indicates that CLBP is processed differently than acute back pain [6]. The present article will review basic biopsychosocial issues impacting chronic low back pain, the use of interdisciplinary programs as an appropriate treatment, and the results of a large prospective series of patients treated with this type of program.

### 1.1. Psychosocial Factors

The role of psychosocial factors has been well documented in both the development of CLBP and the resulting disability. These factors have been found to increase risk for acute low back pain developing into chronic pain, and for increased risk of disability associated with low back pain [7,8,9]. In addition, these factors adversely impact outcomes of both surgical intervention as well as success of interventional technologies such as spinal cord stimulation or intrathecal pumps for pain control [10,11,12,13].

A variety of psychosocial factors have been found to be of relevance [8,14]. Clinical assessment often examines an array of factors [11]: contextual factors such as work related factors, co-occurring life stressors, financial and social reinforcement for pain behavior and disability, and patterns of medical practice, all of which impact outcomes. Other factors evaluated include psychosocial factors such as depression and anxiety, as well as patient expectations, fear of movement (kinesiophobia), reactivity to pain such as pain catastrophizing, and external locus of control. Information is often obtained through both interview and psychometric testing.

In light of data indicating the impact of a biopsychosocial model, interventions based on these factors are used with varying degrees of frequency and have become commonly recommended [15,16]. The most common nonsurgical or non-medication intervention has been physical therapy with most patients being prescribed therapy at some point in their treatment. A second well-established approach has been cognitive behavioral therapy (CBT) to address the psychosocial factors. [17,18].

### 1.2. Interdisciplinary Pain Management Programs

In light of the interactive nature of many factors using the biopsychosocial model, one response has been the development of the interdisciplinary pain management program [19]. To address this complex array of factors, interdisciplinary pain programs began to appear in the United States initially led by John Bonica. Over the last 40 years, such pain programs have been created throughout the United States. These programs have been described by a primary accreditation organization, CARF International. They define a program in this way: “An interdisciplinary pain rehabilitation program provides outcomes-focused, coordinated, goal-oriented interdisciplinary team services”. p.12 [20]. They include goals such as a reduction of impairment and activity limitations, while maximizing quality of life.

These programs include multiple types of providers (physicians, psychologists, nurses, occupational therapists, physical therapists) offering coordinated services “under one roof”, with frequent communication through team meetings around a unified vision and goals. They utilize standardized measurement of functioning of physical ability, pain and suffering, emotional distress, utilization of medical resources, and functional activities of living. The model emphasizes the use of structured supervised physical activation with treatment to change behavioral and social patterns which have evolved from, and have changed, the patients’ experience of pain as well as their life functioning. Treatment is designed to address the array of factors impacting patients’ pain, distress, and subsequent disability. It is interesting that some of these factors are ones found by Pincus [8], which predict chronicity/disability in prospective cohorts with low back pain.

Over the last 30 years, the effectiveness of these programs has been extensively examined for both treatment efficacy and cost-effectiveness [19,21,22,23,24,25,26]. Fullen *et al*. [27] documented effectiveness of treatment of 553 patients with pain over an 8-year period of time. Chou and colleagues in 2009 [28], in the American Pain Society’s Guidelines for Low Back Pain, gave a “strong” recommendation for the use of interdisciplinary treatment and rated evidence as “high” quality. More recently, a Cochrane System Review and meta-analysis found moderate-quality evidence of programs reducing pain and disability [29]. When clinical efficacy and cost efficacy of interdisciplinary pain programs were compared to conventional treatment [23,30], the programs resulted in greater pain reduction, medication reduction, reduction of emotional distress, decreased health care utilization, reduction of iatrogenic consequences, increased activity/return to work, and closure of disability claims. These same reviews compared cost of interdisciplinary programs to surgical intervention, and conventional care including the cost of initial treatment, subsequent surgery, medical treatment in the year following, and lifetime disability. Interdisciplinary treatment was found to be nine times more cost-effective than conservative treatment. Some studies have documented the economic cost of patients being placed on waitlists for interdisciplinary pain facilities [24]. In addition to these group changes, Federoff *et al.* [31] documented the efficacy of programs at the level of individuals, rather just on a group basis. Significant variability was found in response to treatment, but no clear predictors of response were found. In an extension of work by Morley [32], Smith *et al.* [33] repeated an analysis of both group and individual response. They also found that outcomes changed when outcomes from two different time sequences were compared.

Several issues warrant further exploration. First, few studies have examined the impact of providing similar programs at multiple sites to see if effectiveness is similar in various treatment settings. Second, the role of the duration of treatment has been explored in a limited fashion [34,35,36] without evidence of strong effect of duration of treatment. Oslund *et al.* [36] in an unpublished dissertation compared outcomes for patients with three levels of treatment: 120 h, 72 h, and 24 h. It was found that patients with higher dysfunction pretreatment (*i.e.*, greater number of hours resting a day and high levels of pain) profited more with high intensity treatment, whereas persons with lower dysfunction did not respond differentially to levels of intensity of treatment.

In order to further evaluate these issues, the present study utilized a large data set collected over 14 years from two programs with similar models but different locations to address three questions. First, prior to analyzing site and duration, did the program as a whole produce clinically significant change at the completion of treatment across a broad range of outcomes in a large sample of persons with low back pain? Second, were outcomes at the two sites of the program similar or significantly different? Third, did a range of intensity (days of treatment) create differences in outcomes? 

## 2. Experimental Section

Data were collected from a larger data set accumulated for quality control purposes of patients attending the comprehensive interdisciplinary program (Baylor Center for Pain Management) from 2000 to 2014. Four hundred eighty five patients (40%) had a primary diagnosis of back pain according to the Pain Region, as defined by the International Association for the Study of Pain [37]. Of these, 393 (81%) completed treatment. Patients provided written consent to have data included for outcomes analysis. This retrospective study was approved by the IRB at the Baylor Research Institute.

### 2.1. Program Description

The comprehensive outpatient program at the Baylor Center for Pain Management in Dallas, Texas, has provided interdisciplinary treatment since 1995. This program was carried at two sites—in Dallas and Richardson—with the same treatment protocol, staffing patterns, program direction, and standard outcomes measures and procedures. Each site utilized a psychologist, a licensed professional counselor providing biofeedback, an occupational therapist, a physical therapist, and a case manager. Both sites were under the direction of the same program director over the 15 years providing broad stability in treatment and data collection. Program size would range from 2–12 patients at a time. Patients were included if pain was chronic (over 6 months), it interfered with functional activities and/or demonstrated elevated emotional distress, and patients reported desire for improvement and functional goals. Patients were excluded if their level of psychiatric problems or cognitive functions would interfere with participation and understanding in a group-oriented program. As an outpatient program, patients were required to be safe and independent for all self-care, ambulation, and transfers. Patients were also required to set goals and to commit to regular participation in the programs.

Initially, the program consisted of 20 days of treatment (approximately 100 h) but, over time, has been reduced to 12 days of treatment (approximately 60 h) to adapt to scheduling needs of patients and insurance carriers. Daily patients participated in one hour of physical therapy, one hour of aquatics therapy, one hour of counseling or biofeedback, one hour CBT group, one hour occupational therapy group, one mind/body technique for pain control, and one hour occupational therapy with focus on lifestyle management for adaptive living including return to work. Training in meditative practice, biofeedback, as well as consultation with chaplain services and nutrition services were included.

### 2.2. Measurement

Data were gathered upon admission to the program (Day 1) and at the final day of treatment. Outcomes included changes in pain, emotional distress, activity levels, medical utilization, physical abilities, instrumental activities of living, and patients’ perception of change. These data are used to measure primary outcomes indicated by the IMPACCT group [16,38].

#### 2.2.1. Descriptive Statistics

Upon admission, patients completed a questionnaire which included demographic data, duration of pain (in months), and estimate of utilization of medical resources in the year prior.

#### 2.2.2. Outcome Measures

Abbreviations are as follows: the Multidimensional Pain Inventory (MPI) [39]; Beck Depression Inventory (BDI); Canadian Occupational Performance Measure (COPM) [40]. Daily Life Questionnaire measured patient rating of hours active, efficacy of non-medication pain management techniques, and physical therapy including distance walked in 5 min (measured in laps).

#### 2.2.3. Patients’ Rating of Change

In addition, at graduation, patients completed the Patients’ Global Impression of Change (PGIC) scale [41] and a rating of changes in use of medication.

### 2.3. Data Analysis

Initial analysis was made by combining data from graduates from the two sites. Descriptive analysis was conducted for the program’s primary descriptors of the participants, including frequency of categorical variables and means and standard deviations for continuous variables with the exception of duration for which median and range were chosen due to the extreme variability. Shapiro-Wilk tests for normality of distribution were carried out on variables. Because distributions did not meet assumptions of normality, a series of Wilcoxon signed rank tests were used to measure the effects of treatment. Effect size was measured using the value of r, which is appropriate for Wilcoxon tests.

Second, outcome variables from the two sites were carried out using repeated-measures ANOVAs, with treatment site as one variable and individual patient’s change in scores from pre to post as the second variable. It should be noted that, although some measures were not normally distributed, ANOVAs are robust to minor deviations from normality. Chi-square tests were used for categorical variables, such as the Patient’s Global Impression of Change.

Third, the relationship between the numbers of days of treatment and the amount of change was calculated using a series of correlation coefficients.

## 3. Results and Discussion

### 3.1. Descriptive Data from Both Sites

Analysis of descriptive statistics (see Table 1 and Table 2) found significant differences on almost all variables except gender distribution, suggesting that populations were somewhat different. Patients at Site 1 were significantly (*t*(392)= −2.78, *p* = 0.004) younger (mean = 51.8, SD = 12.4) than those at Site 2 (mean = 56.6, SD = 12.0). The duration of pain in months was also significantly shorter (*t*(354)= 3.0, *p* = 0.002) in Site 1 (mean = 95.6, SD = 117.1) than in Site 2 (mean = 146, SD = 138.9), although the distribution was so broad as to undermine meaningfulness of central tendency. Gender distribution was not different (chi square = 0.38, *p* = 0.53). Payer source was also significantly different (chi square = 11.09, *p* = 0.01).

### 3.2. Outcomes from Combined Sites

Seven standard measures (see Table 3) were used to determine outcome by comparing scores from the start of the program (pre) to the completion of the program (post). None of the seven measures met assumptions for the normality of distribution as measured by the Shapiro–Wilk test. Paired sample *t*-tests found that changes for all variables were significant at the 0.001 level using Wilcoxon signed rank *t*-tests.

These changes were found for both self-report repeated measures—self-reported perception of clinical progress and objective measures obtained by a physical therapist (laps walked in 5 min). Changes were large as evidenced by effect sizes (ES’s), ranging from 0.66–0.85. All changes were at *p* = 0.001.

#### 3.2.1. Pain

Although only 36% of patients (see Table 4) achieved a pain severity reduction of 30% as a recommended target, [42], change was significant as demonstrated by a a large ES (0.6), and the helpfulness of nonmedical techniques used to manage pain had improved by 129% (ES = 0.85). In addition, it has been noted that pain reduction alone may not be an appropriate primary goal for chronic back pain [43].

#### 3.2.2. Functional Activities

Prominent changes in function were also found. Interference by pain was reduced by at least 30% for 47% of patients (ES = 0.77), 70% of patients increased hours of activity by 30% (ES = 0.78), and 91% of patients increased by at least 30% in their ability to carry out primary desired activities of living as measured by the COPM (ES = 0.85).

#### 3.2.3. Physical Measures

These self-reported changes in activity and functional ADLs were mirrored by changes in physical functioning. Distance walked in a 5-min timed task improved by at least 30% in 61% of patients (ES = 0.85).

#### 3.2.4. Psychological Functioning

Ratings of emotional distress markedly improved. Using standard interpretation of scores, depression decreased by at least 30% in 78% of patients (ES = 0.82) with mean scores changing from moderate (pre-treatment mean = 21.5) to minimal (post-treatment mean = 10.2) range of depression using standard interpretation of the scores.

#### 3.2.5. Medication Use

Due to the challenges in quantifying medication usage in this clinical setting, data were not collected on specific types or dosages of medication used by each patient. However, patients were asked at graduation to report any change in the use of pain medication. Of the 344 patients in this group: 13 (9%) reported taking no medications; 162 (47%) reported taking fewer; 138 (40%) were unchanged in the use of medication; and 31 (9%) reported taking more medication.

#### 3.2.6. Patient Rating of Change

Patients reported positive changes on global impression of change. Eighty-two percent of 281 patients reporting met the *a priori* goal of either “much improved” (n = 103, 36%) or “very much improved (n = 131, 46%)”. Forty-four (15%) reported “minimal improvement”, and only 3 (1%) reported “no change”. No patients reported being “minimally worse,” “much worse”, or “very much worse”.

### 3.3. Comparison of Outcomes from the Two Sites

It should be noted that, although some measures were not normally distributed, it was decided that parametric tests would be conducted because of the clarity of presentation, as well as the fact that ANOVAs are quite robust to minor deviations from normality. Therefore, repeated-measures ANOVAs (see Table 5) were conducted for a subset of outcomes chosen from various domains using the site as one variable and the change in repeated measures as another. Three analyses were conducted. Site outcomes were similar in most variables. Overall scores, combining pre- and post-scores at the two sites were similar with differences in only 2 of 7 measures: interference by pain (F = 9.46, *p* = 0.002) and distance walked in five minutes (F = 24.56, *p* = 0.001). Second, as reviewed above in Table 4, when sites were combined, the comparison of individuals pre/post measures were different at the *p* < 0.05 level and, in fact, were at the *p* = 0.001 level.

Some variability was found between measures in the interaction of the site and outcome measures. For four of the seven measures (pain intensity, pain interference, helpfulness of pain techniques, depression, satisfaction with ADL), the interaction of Site × Change was not significant at the *p* = 0.05 level. Hours of activity and distance walked each demonstrate an interaction effect of Site × Change and an amount of change (F = 406.8, *p* = 0.009; F = 7.62, *p* = 0.006). Chi-square tests of patient ratings of five ratings of improvement by pain site revealed no significant difference between sites (chi square = 4.620, *p* = 0.33).

### 3.4. Impact of Intensity of Treatment with Outcomes

Finally, the amount of change for selected variables was evaluated to determine correlation with the number of days of treatment completed. Although several change scores were correlated at the *p* = 0.01 level (*i.e.*, helpfulness of techniques) and at the *p* = 0.001 level (*i.e.*, hours active, distance walked), these correlations were quite low. For example, although significant, the number of days of treatment completed only accounted for 4% of the variance (*r*^2^ = 0.41) in the change in hours active per day.

## 4. Conclusions

This study explored three aspects of interdisciplinary programs for treatment of chronic pain. In the first analysis, results were consistent with previous literature indicating that interdisciplinary pain management programs can provide broad-based change, even for patients with entrenched CLBP associated with high pain, emotional distress, functional disability, and excessive use of medications and medical resources. Consistent with recommendations by the Initiative on Methods, Measurement, and Pain Assessment in Clinical Trials (IMMPACT) group and others for research with chronic low back pain [38,44], it included repeated measures of pain, physical functioning, emotional functioning, and participants ratings of improvement. These changes were both highly significant statistically, as well as having large effect sizes. These findings are consistent with the previous studies of the effectiveness of interdisciplinary pain programs for patients with low back pain [22,23]. Of note, the types of change and size of change are comparable to those obtained with more invasive or intensive medical measures, which are more costly or have higher iatrogenic risks. Interdisciplinary pain programs have produced an analgesic effect comparable to opioids for CLBP or back pain with chronic radiculitis, but without the risk of overdose, addiction, and diversion [45,46]. The functional benefits noted are also important because they are not associated with the risks and complications associated either with spine surgery or even spinal cord stimulation [19].

Although this study was confirmatory of previous studies, a number of potential limitations should be noted. First, this was not a randomized controlled trial, but data were collected on a prospective basis as part of a quality assurance assessment. As such, there was no control group, so a potential placebo effect could not be measured or ruled out. Second, 20% of patients discontinued treatment for a variety of reasons, and, due to the focus of this study, no analysis was made of factors predicting non-completion or the degree of change during the program. A previous study of these programs, however, with all diagnostic groups included, found predictors for non-completion and identified factors predicting a response to treatment [36].

In addition, these changes reflect immediate effects of treatment at program completion. Additional literature [31,32,33] suggests that the psychological effects of intervention tend to lessen over time. This was found in an earlier study with this same program [47]. Continued change from pre-treatment was found on all measures at the *p* > 0.001 level, both six months and one year following treatment. However, six months later, some measures had regressed towards the pre-treatment scores, yet others had remained stable, and some had even continued to improve. All measures had regressed somewhat one year following treatment.

In the second analysis, this study also documented that interdisciplinary programs were effective across treatment sites. Patient populations were somewhat different based on descriptive data, such as age and duration of pain. Despite this, groups did not differ globally on a variety of measures selected to represent an array of domains. In addition, there were no differences in change at the two sites on six of seven measures, although difference in response was noted on one measure: change in distance walked in five minutes. This study suggests that, even with somewhat different populations, interdisciplinary programs were robust in the change they produced.

In the third analysis, this study also provided evidence that change was only minimally correlated with the number of days of treatment. Despite statistically significant correlations between the numbers of days of treatment, days of treatment only accounted for 4% of variance at most. This finding is consistent with the results of a previous review [35].

As Gatchel *et al.* [19] noted in their recent review, it is troubling that interdisciplinary pain programs have often been allowed to fail. It continues to be puzzling that a treatment protocol with well-documented improvements in patient quality of life and patient satisfaction is so often unavailable or not offered to patients (and healthcare providers) who are “stuck”. The benefit to healthcare systems would likely be even greater if treatment programs were provided to patients much sooner following the onset of pain. This study provides additional confirmation that these programs may robustly provide effective treatment, even with variation between program staff and settings and with variable duration of treatment.

## Figures and Tables

**Table 1:** Descriptive statistics of each sample site. (Group 1 = Dallas; Group 2 = Richardson).

Variable	Group	N	Mean	SD	SEM
Age	1	326	51.877	12.377	0.686
	2	68	56.603	12.019	1.457
Duration of pain (months)	1	291	95.680	117.109	6.865
	2	65	146.400	138.923	17.231
Pain Treatment in Last Year (Mean/SD)					
# of MD Office visits due to pain	1	317	0.934	0.775	0.044
	2	67	0.955	0.475	0.058
# of mental health visits for pain	1	300	3.063	7.091	0.409
	2	67	3.836	7.168	0.876
# of ED visits for pain	1	298	0.977	1.704	0.099
	2	68	0.500	1.029	0.125
# of diagnostic procedures for pain	1	306	2.278	1.867	0.107
	2	65	1.815	1.776	0220
# of treatment procedures for pain	1	311	2.103	2.194	0.124
	2	67	2.836	5.918	0.723
# of surgeries for pain	1	308	0.786	1.377	0.078
	2	67	0.433	0.857	0.105

Note: SEM: Standard error, SD = standard deviation, MD = Visits to a physician, ED = Visits to the emergency department.

**Table 2:** Additional descriptive statistics (categorical) of each sample site. (Group 1 = Dallas; Group 2 = Richardson).

Payer Source	Group	N	Percent
Worker’s Comp	1	44	15.1
	2	1	1.5
Commercial	1	142	48.8
	2	36	53.7
Medicare	1	100	34.4
	2	30	44.8
Others	1	5	1.7
	2	0	0.0

**Table 3:** Comparison of pre- and post- program treatment outcomes using Wilcoxon signed rank tests.

Variable	Pretreatment Mean + SEM	Posttreatment Mean + SEM	Statistic (df)	*p* Value	Effect Size *
*Pain severity* (*n* = 375)	8.61 ± 0.10	6.74 ± 0.11	45,498 (374)	<0.001	0.66
*Pain interference* (*n* = 375)	10.18 + 0.10	7.21 + 0.15	48,217 (374)	<0.001	0.77
*Depression* (*n* = 376)	21.52 + 0.54	10.18 + 0.44	63,676 (375)	<0.001	0.82
*Hours active* (*n* = 360)	4.88 ± 0.15	8.26 ± 0.15	2725 (357)	<0.001	0.78
*Helpfulness* (*n* = 341)	3.28 + 0.11	7.53 + 0.10	509 (340)	<0.001	0.85
*Ability to do ADLs* (*n* = 259)	3.35 + 0.12	7.50 + 0.32	252 (258)	<0.001	0.85
*Distance walked* (*n* = 377)	14.17 + 0.28	20.49 + 0.33	701.97	<0.001	0.85

Pain severity, Pain Interference, (Modified Multidimensional Pain Inventory—Pain Severity, Pain Interference, Range = 0–12), Depression (Beck Depression Inventory-II), Helpfulness (How helpful are your techniques to manage your pain? Range 0–10), Ability to do ADLs (Canadian Occupational Performance Measure), Distance walked (laps walked in 5 min). * The r statistic (the z score divided by the square root of the number of observations) was used for determining Effect Size): Small ES = 0.10, Medium = −0.30, Large = 0.50.

**Table 4:** Percent of patient’s achieving 30% or greater change.

Variable	# Achieving 30%/Total N	Percent Achieving 30%
Pain severity	136/375	36%
Pain interference	177/375	47%
Depression	296/376	78%
Hours active	251/360	70%
Helpfulness	278/341	82%
Ability to do ADLs	236/259	91%
Distance walked	231/377	61%

Note: # = Number.

**Table 5:** Changes in outcome variables with 95% confidence intervals for both groups (program sites), within subjects (pre/post differences for combined sites), and interaction of group (program site by within subjects).

Variable	Site 1	Site 2	Between Group	Within Subjects	Within Sub Group
*Pain severity*			F = 2.07, *p* = 0.15	F = 103.5, *p* = 0.001	F = 1.90, *p* = 0.16
Pre-program	8.7 (2.0)	8.1 (1.9)			
Post-program	6.8 (2.2)	6.7 (2.6)			
*Pain interference*			F = 9.46, *p* = 0.02	F = 219.80, *p* = 0.001	F = 3.68, *p* = 0.06
Pre-program	10.3 (2.0)	9.8 (2.3)			
Post-program	7.4 (2.9)	6.1 (3.4)			
*Helpfulness of Pain Techniques*			F = 1.49, *p* = 0.22	F = 863.1, *p* = 0.000	F = 2.78, *p* = 0.09
Pre-program	3.3 (2.1)	3.7 (2.4)			
Post-program	7.5 (2.0)	7.5 (1.8)			
*Hours active*			F = 0.00, *p* = 0.98	F = 406.8, *p* = 0.000	F = 6.84, *p* = 0.009
Pre-program	4.7 (2.8)	5.4 (7.8)			
Post-program	8.3 (2.9)	7.8 (2.8)			
*Depression*			F = 2.03, *p* = 0.15	F = 306.8, *p* = 0.001	F = 0.210, *p* = 0.65
Pre-program	21.7 (10.4)	20.4 (11.5)			
Post-program	10.5 (8.8)	8.6 (7.9)			
*Distance Walked*			F = 24.56, *p* = 0.001	F = 323.4, *p* = 0.001	F = 7.62, *p* = 0.006
Pre-program	14.7 (5.7)	11.9 (4.0)			
Post-program	21.3 (6.5)	16.8 (4.5)			
*Performance of ADLS*			F = 0.69, *p* = 0.79	F = 147.9, *p* = 0.000	F = 1.10, *p* = 0.315
Pre-program	3.3 (2.2)	3.6 (1.5)			
Post-program	7.6 (5.9)	7.1 (1.6)			

Pain severity, Pain Interference (Modified Multidimensional Pain Inventory—Pain Severity and Pain Interference scales, Range = 0–12), Depression (Beck Depression Inventory-II), Helpfulness (How helpful are your techniques to manage your pain? Range 0–10), Performance of ADLs (Canadian Occupational Performance Measure), Distance walked (laps walked in 5 min)).
